# Low-Dose Intravenous Methylprednisolone in Remission Induction Therapy for ANCA-Associated Vasculitis

**DOI:** 10.34067/KID.0000000000000222

**Published:** 2023-09-05

**Authors:** Lauren Floyd, Adam D. Morris, Anamay Shetty, Mark E. Brady, Arvind Ponnusamy, Paul Warwicker, Ajay Dhaygude

**Affiliations:** 1 Division of Cardiovascular Sciences, University of Manchester, United Kingdom; 2 Department of Renal Medicine, Lancashire Teaching Hospitals NHS Foundation Trust, Preston, United Kingdom; 3 University of Cambridge, Cambridge, United Kingdom

**Keywords:** ANCA, ANCA-associated vasculitis, glomerulonephritis, glucocorticoid toxicity, steroids, methylprednisolone, vasculitis, glomerular and tubulointerstitial diseases, immunosuppression

## Abstract

**Key Points:**

- The contribution of IV methylprednisolone to glucocorticoid toxicity is often overlooked with limited evidence supporting its use.
- Markedly reduced cumulative glucocorticoid dosing for remission induction therapy in AAV is safe and effective.
- Reduced IV methylprednisolone and radical steroid avoidance strategies have not been shown to have any significant adverse effect on outcomes.

**Background:**

Glucocorticoids (GCs) remain integral to the management of ANCA-associated vasculitis (AAV), but are associated with significant adverse effects. Recent studies have shown reduced oral GC dosing to be safe and effective; however, data guiding the use of intravenous (IV) methylprednisolone (MTP) are limited.

**Method:**

A single-center retrospective cohort of patients with AAV were divided into two groups: low-dose GC (patients receiving 250 mg of IV MTP, followed by a tapering course of 30 mg of prednisolone daily) versus high-dose GC (1.5 g of IV MTP, followed by a tapering course of 40–60 mg of prednisolone daily). Primary outcomes included ESKD and mortality, and secondary outcomes included GC-related toxicity, remission, and relapse rates. This study was applied to patients with newly diagnosed AAV, including those with severe or life-threatening disease.

**Results:**

Sixty-five patients were included in the final analysis—34 in the high-dose treatment group and 31 in the low-dose treatment group. At diagnosis, more advanced renal impairment and histological disease were present in the low-dose cohort. The rate of ESKD was similar between the groups at 6 and 12 months (*P* = 0.22, *P* = 0.60, respectively). More deaths occurred in the high-dose group (26.5% versus 6.5%, *P* = 0.05), although this was not significant on multivariable analysis (*P* = 0.06). Remission rates were comparable, and there was no significant difference in relapses. Adverse events were seen in both groups, but patients in the high-dose group experienced a higher incidence of severe infections, weight gain, and steroid-induced diabetes.

**Conclusion:**

We demonstrate that a markedly reduced dose of IV MTP with a lower overall cumulative dose of GCs is safe and effective in the management of severe AAV disease, with no significant difference in primary outcomes.

## Graphical abstract

**Figure:**
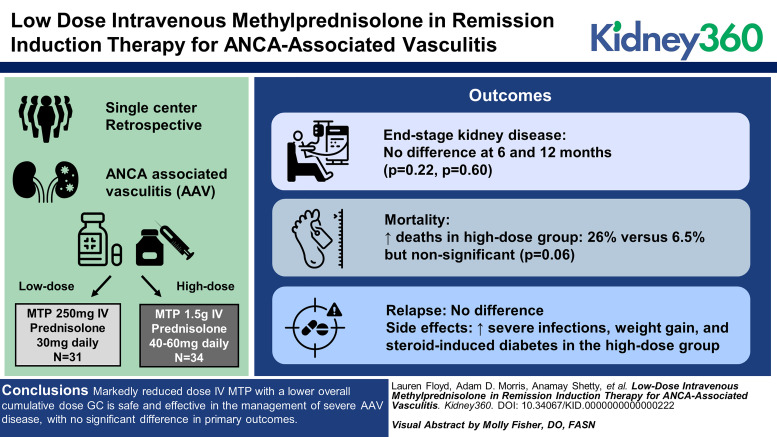

## Introduction

ANCA-associated vasculitis (AAV) is a rare and complex autoimmune disease characterized by inflammation and necrosis of small blood vessels. Over the past three decades, advancements in treatment strategies have reduced AAV mortality, transforming it into a chronic relapsing–remitting disorder associated with accumulating morbidity. The cause of this is multifactorial, resulting from disease-associated damage, increased cardiovascular risk, infection, and treatment-related toxicity, of which glucocorticoid (GC) exposure is a key factor.^1–4^

**Figure 1:**
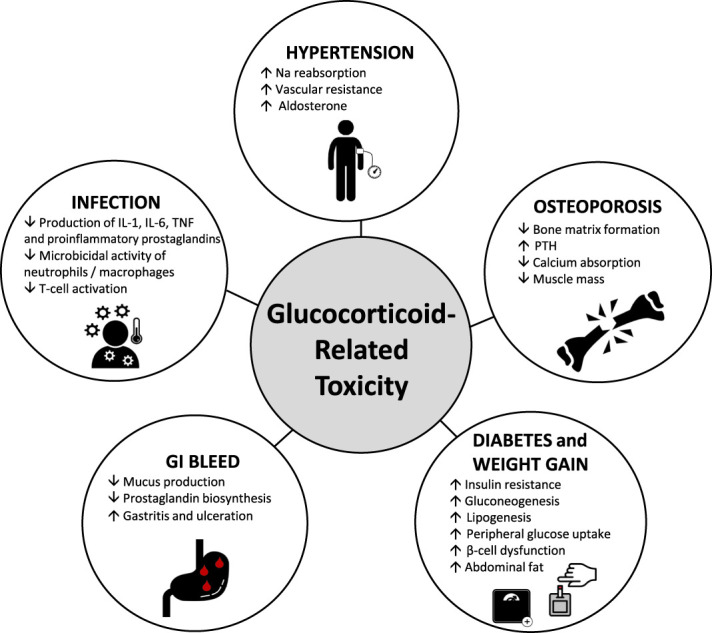
**Pathophysiological processes involved in glucocorticoid-related toxicity.** GI, gastrointestinal; Na, sodium; PTH, parathyroid hormone; TNF, tumor necrosis factor; IL, interleukin.

Both remission induction and maintenance therapies for severe disease typically use tapering doses of GCs alongside cytotoxic immunotherapy with cyclophosphamide and/or B-cell depletion with rituximab.^5^ The adverse effects of GCs in the AAV patient population are well-recognized, and the development of the Glucocorticoid Toxicity Index^6^ has provided a quantifiable assessment tool used to demonstrate the direct detrimental effect that cumulative GC exposure has on patients^1,2,7^ (Figure 1) While GCs remain the cornerstone of treatment, it is only in recent years that the toxic side effects have been considered and quantified as outcomes.

Despite these advancements and the recognized associated risk with a higher cumulative dose of GCs, there is comparatively very limited guidance regarding the role of intravenous (IV) methylprednisolone (MTP) as part of remission induction therapy.^8^ Current mainstream practice evolved from the late 1970s, a time when there were very few steroid-sparing treatment options. There has been limited evaluation of its role and dosing strategy since. This study aims to explore the role of high versus low-dose IV MTP as part of standard remission induction therapy.

## Methods

### Participants and Study Design

A cohort study of patients with newly diagnosed AAV from a single center in the United Kingdom was conducted between 2007 and 2022. All patients had kidney disease with a diagnosis of AAV in accordance with the Chapel Hill consensus.^9^ Patients with severe or life-threatening disease, including those presenting with rapidly progressive glomerulonephritis, serum creatinine >350 *µ*mol/L, and/or diffuse alveolar hemorrhage, were included. Any patient with seronegative disease required confirmation of disease on renal biopsy.

In December 2018, a departmental protocol introduced a new lower dosing regimen of IV MTP and oral GCs as standard remission induction treatment, for all patients presenting with organ or life-threatening AAV disease. The majority of patients presenting after this date were treated with low-dose GCs, defined as a single dose of 250 mg pulsed IV MTP, followed by a tapering course of oral prednisolone starting at 30 mg/d. These patients were compared with a historic cohort of patients with AAV who received higher dose GC therapy, defined as 1.5 g of IV MTP given over 3 consecutive days, followed by a tapering course of oral prednisolone starting at 40–60 mg/d. This was in keeping with what most physicians would have considered the standard of care at the time. Patients who received any deviation from the outlined treatment doses described above or where adherence to them could not be confirmed from retrospective data were excluded from the study, to ensure robust comparison of the two steroid treatment arms. Other exclusion criteria included patients with dual positivity for ANCA and anti-glomerular basement membrane antibodies, as well as those with secondary vasculitis, aged <18 years and <3 month follow up data.

The oral GC regimens are summarized in Supplemental Table 1. Cumulative doses of GCs were calculated at 3, 6, and 12 months. Pulsed IV MTP doses at induction were converted to oral prednisolone dose equivalents, with all subsequent cumulative doses presented as prednisolone (mg). Alongside GCs, remission induction regimen included cyclophosphamide, rituximab, or combination therapy in line with the dosing protocol applied in the Rituximab versus Cyclophosphamide in ANCA-Associated Vasculitis trial.^10^ Choice of induction regimen was based on physician discretion. The dosing regimen of IV cyclophosphamide was adjusted for age and renal function in accordance with the European Vasculitis Study Group.^11,12^ Rituximab therapy was administered at a dose of 1 g fortnightly for a total of two doses.

Disease remission was defined as a Birmingham Vasculitis Activity Score of less than one. Disease relapse was defined as deterioration in signs and symptoms attributable to vasculitis requiring escalation of immunosuppressive medication. The following data were collected retrospectively from the time of diagnosis: demographics; Charlson Comorbidity Index (CCI) at presentation^13,14^; clinical presentation; immunotherapy; patient outcomes; laboratory investigations; histopathology data, including Berden classification^15^; and Renal Risk Score (RRS).^16^

This study met the criteria for service evaluation; therefore, formal ethics committee review was not required. All patients consented to the treatment protocol agreed upon at our center, and the study was performed in accordance with the Declaration of Helsinki.

### Outcomes

The primary outcomes were mortality and ESKD at the end of follow-up, which was a minimum of 3 months from diagnosis. ESKD was defined as dialysis dependence for over 90 days or kidney transplantation. Secondary outcomes included remission rates, time to remission, relapse rates, and serious adverse events. Serious adverse events were defined as infections requiring hospitalization, bone marrow failure, fragility fractures, new diagnosis of malignancy, cardiovascular events, gastrointestinal bleeding, and steroid-induced diabetes mellitus.

### Statistical Analyses

We performed basic descriptive statistics for the outcome variables and confounders. Using linear models, we analyzed the difference in outcomes between the two groups according to IV MTP dosing 1.5 g versus 250 mg. Continuous outcomes were modeled using standard linear regression and dichotomous outcomes using a logistic regression model. 1.5 g was used as the baseline group, and the coefficient associated with the treatment variable was interpreted as the effect of 250 mg on the outcome in question, with an associated *P*-value. *P*-values were further interpreted on the basis of Bonferroni correction for multiple hypothesis testing. In addition to the unadjusted model described above, we generated linear models which were controlled for comorbid scores, age, sex, and immunosuppressive therapy. We used multiple linear regression to control for confounders. A linear mixed model, with varying intercepts for each patient and a fixed effect for treatment, was used to estimate the effect of treatment on eGFR over time.

## Results

In total, 173 patients were treated with IV MTP between 2007 and 2022. Of these, 34 received high-dose GC induction treatment (1.5 g of IV MTP over 3 days, followed by oral prednisolone starting at 40–60 mg/d) and 31 received the low-dose regimen (single dose of 250 mg of pulsed IV MTP, followed by oral prednisolone starting at 30 mg/d). One hundred eight patients were excluded because of variations in the GC dosing that fell outside the inclusion criteria or where there was uncertainty in the retrospective dosing data. All those who received low-dose GC were treated after December 2018, and most of the patients who received the higher dose regimen were treated between 2007 and 2018.

The demographics of the two cohorts are presented in Table 1, alongside the clinical features and extrarenal organ involvement. Across the cohort, there was a male predominance; in particular, there were a higher number of men in the high-dose cohort (73.5% versus 26.5%). The CCI^13^ was completed for all patients, with fewer comorbidities observed in the low-dose cohort (mean CCI of 3.6 versus 4.6). At the time of diagnosis, both cohorts had a similar degree of renal impairment and nearly one-third of patients in each cohort required renal replacement therapy at presentation—32.4% (*n*=11) in the high-dose cohort and 32.3% (*n*=10) in the low-dose cohort. Most of the patients (*n*=56) underwent a renal biopsy, which confirmed pauci-immune glomerulonephritis. Nine patients were treated without a histological diagnosis. In three patients, this was because of a high risk of bleeding, and the other six patients were considered frail with a classical presentation and high ANCA titers, consistent with a diagnosis of AAV.

**Table 1:** Demographics and disease burden between patients who received high-dose glucocorticoids (1.5 g of intravenous methylprednisolone over 3 days, followed by a tapering course of oral prednisolone starting with 40–60 mg/d) versus low-dose glucocorticoids (single dose of 250 mg of intravenous methylprednisolone, followed by a tapering course of oral prednisolone starting with 30 mg/d)

Characteristics	Total (*N*=65)	High-Dose GC (*n*=34)	Low-Dose GC (*n*=31)
**Patient Demographics**			
Age, mean (SD), yr	69.2 (10.4)	69.1 (9.3)	69.3 (11.6)
Sex (M:F)	37:28	25:9	12:19
Charlson Comorbidity Index	4.1	4.6	3.6
Creatinine at presentation, umol/l, median (IQR)	422 (214–630)	406 (209–640)	422 (215–551)
eGFR at presentation, ml/min Median (IQR)	10 (7–24)	11 (6–26)	10 (7–20)
RRT at presentation, *n* (%)	21 (32.3)	11 (32.4)	10 (32.3)
Pulmonary hemorrhage, *n* (%)	10 (15.4)	7 (20.6)	3 (9.7)
**ANCA subtypes, (*n*)**			
MPO	36	19	17
PR3	28	14	14
Negative	1	1	0
**Clinical features and organ involvement, *n***			
Renal limited	16	8	8
Constitutional symptoms	28	13	15
Pulmonary	31	13	18
ENT	15	7	8
CNS	9	7	2
Cutaneous	13	7	6
Ophthalmic	6	3	3
Gastrointestinal	3	3	0

GC, glucocorticoid; M, male; F, female; IQR, interquartile range; RRT, renal replacement therapy; MPO, myeloperoxidase; PR3, proteinase-3; ENT, ears, nose, and throat; CNS, central nervous system.

### Treatment

Remission induction immunosuppression with cyclophosphamide and/or rituximab was given to all patients (Supplemental Table 2). IV cyclophosphamide monotherapy was used in 39 patients—25 in the high-dose group and 14 in the low-dose group—with a mean cumulative dose of 5.3±2.8 g. Rituximab monotherapy (total 2 g) was given to 11 patients: three in the high-dose group and eight in the low-dose group. A combination of low-dose cyclophosphamide and rituximab (2 g) was used in 15 patients: six in the high-dose cohort and nine in the low-dose cohort; among these, the mean dose of cyclophosphamide was 2 g. One patient in each cohort received a higher dose of cyclophosphamide (3.6 and 4.3 g) in addition to rituximab because of disease severity with pulmonary hemorrhage. Plasma exchange was used similarly across both cohorts, and the mean number of sessions was 6±2.

Daily and cumulative doses of GC were reviewed at 6 and 12 months from diagnosis (Supplemental Table 2). There was a significant difference in the daily GC dosing between the high and low-dose cohorts at 6 and 12 months (*P* = 0.01 and *P* = 0.02). There was also a significant difference in the mean cumulative doses at 3, 6, and 12 months between the two groups after Bonferroni correction (*P* < 0.001).

At the time of follow-up, most of the patients (*n*=60, 92.3%) were receiving maintenance therapy. Of the five patients who did not receive maintenance immunosuppression, two died, two were lost to follow-up, and one remained dialysis-dependent with no extrarenal involvement. The maintenance treatment was comparable across the high and low-dose groups, with most of the patients receiving either azathioprine (11 and 14, respectively) or rituximab (17 and 16, respectively). Prophylactic co-trimoxazole and fluconazole were used alongside bone and gastric protective treatments in all patients.

### Primary Outcomes

Berden classification^15^ and RRS^16^ were applied, as summarized in Table 2. Berden focal classification was the most common in the high-dose GC cohort (*n*=16), whereas crescentic classification was more common in the low-dose GC cohort (*n*=11). On applying the RRS to predict ESKD, the low-dose GC cohort had a high burden of kidney disease at presentation with fewer normal glomeruli (33.3% versus 48.2%) and a higher number in the high-risk category.

**Table 2:** Histopathological findings between the low-dose and high-dose glucocorticoids groups

Histological Findings	Total (*N*=56)	High-Dose GC (*n*=30)	Low-Dose GC (*n*=26)
**RRS**			
Normal glomeruli (%)	41.3	48.2	33.3
Low risk	9	6	3
Intermediate risk	31	18	13
High risk	16	6	10
**Berden Classification, *n* (%)**			
Focal	23 (41.0)	16 (53.3)	7 (26.9)
Mixed	10 (17.9)	3 (10.0)	7 (26.9)
Crescentic	20 (35.7)	9 (30.0)	11 (42.3)
Sclerotic	3 (5.4)	2 (6.7)	1 (3.9)

GC, glucocorticoid—low-dose GC (250 mg of IV methylprednisolone, followed by a tapering course of 30 mg of prednisolone daily) versus high-dose GC (1.5 g of IV methylprednisolone, followed by tapering course of 40–60 mg of prednisolone daily); RRS, Renal Risk Score^16^ comprising eGFR (G0 >15 ml/min per 1.73 m^2^, G1 ≤15 ml/min per 1.73 m^2^), percentage of normal glomeruli (N0 >25%, N1 10%–25%, N2 ≤10%), tubular atrophy, and interstitial fibrosis (T0 ≤mild to moderate, T1 ≥moderate), with points assigned (G1=3, N1=4, N2=6, T1=2) and risk groups; low 0, moderate 2–7, and high 8–11 points.

The number of patients with ESKD was similar between the groups at 6 months and at the end of the study, with no overall significant difference (*P* = 0.22 and *P* = 0.60, respectively), as presented in Table 3. There were a total of 11 deaths, with 3 deaths occurring in the first 12 months—two in the high-dose cohort and one in the low-dose cohort. The overall mortality rate at the end of study was higher in the high-dose group (*n*=9, 26.5%) in comparison with the low-dose group (*n*=2, 6.5%), although this was not statistically significant when adjusting for follow-up, immunosuppressive therapy, and comorbidities. Where the cause of death was known, two patients died from sepsis, two from severe acute respiratory syndrome coronavirus 2 infection, one from pulmonary hemorrhage, and one because of a myocardial infarction.

**Table 3:** Primary and secondary outcomes associated with high and low-dose glucocorticoids

Outcomes	Total (*N*=65)	High-Dose GC (*n*=34)	Low-Dose GC (*n*=31)	*P* Value
Mortality at 12 mo, (*n*)	3	2	1	0.62
Mortality at EOS, (*n*)	11	9	2	0.05
ESKD at 6 mo, (*n*)	20	8	12	0.22
ESKD at 12 mo, (*n*)	22	10	12	0.52
ESKD at EOS, (*n*)	23	11	12	0.60
RRT at presentation, (*n*)	21	11	10	0.99
Time to remission, d (SD)	113±50	127±58	97±33	0.02
Relapse, (*n*)	14	10	4	0.11
Glucocorticoid-related toxicity, (*n*)	24	15	9	0.31
Infection, (*n*)	7	6	1	0.09
Steroid-induced diabetes, (*n*)	6	4	2	0.47
Reduced bone mineral density, (*n*)	9	5	4	0.83
Weight gain (kg)	+1	+2	+0.2	0.35

GC, glucocorticoids—low-dose GC (250 mg of IV methylprednisolone, followed by a tapering course of 30 mg of prednisolone daily) versus high-dose GC (1.5 g of IV methylprednisolone, followed by a tapering course of 40–60 mg of prednisolone daily); EOS, mo, months; EOS, end of study; d, days; SD, standard deviation.

While the combination of low-dose cyclophosphamide (<2 g) alongside rituximab therapy was not felt to have any significant steroid-sparing effects, multivariable analysis was applied to adjust for those who received dual therapy and there was no significant difference in ESKD or mortality between the groups (*P* = 0.60 and *P* = 0.06, respectively).

### Secondary Outcomes

The length of follow-up between the two groups differed, with a longer duration of follow-up in the high-dose group (1874±1102 versus 419±272 days). Remission was achieved in all high-dose patients (*n*=34) and 96.8% (*n*=30) of the low-dose patients. Time to remission was nominally significantly different between the two groups, with a shorter time to remission observed in the lower dose cohort (97 versus 127 days, *P* = 0.015). This remained significant (*P* = 0.018) in the multivariate analysis when adjusting for additional immunosuppressive therapy and comorbidities, although it is worth noting that the retrospective application of Birmingham Vasculitis Activity Scores in some of the historic, high-dose cohort may have acted as a confounder. There was no difference in relapse rates (*P* = 0.11) or time to relapse (*P* = 0.16) between the groups.

There were a greater number of GC-related toxic adverse events in the high-dose cohort (44% [*n*=15] versus 29% [*n*=9]), although this was not statistically significant (*P* = 0.31) (Table 3). Patients in the high-dose group had an average weight gain of 2 kg from diagnosis to 3–6-month follow-up (average weight increased from 77.4 to 79.4 kg), in contrast to the low-dose group who had only a 0.2-kg cumulative weight gain (average weight increased from 78.6 to 78.8 kg) (*P* = 0.35). Of those who had cumulative weight gain, a similar number in each cohort were on dialysis (5 in each group), in which fluid retention may have contributed to weight gain rather than a true change in body composition.

Six patients in the high-dose group, compared with only one in the low-dose group, developed severe infection requiring hospital admission. Other adverse events included six cases of bone marrow suppression and pancytopenia related to immunosuppressive treatments and one event of cyclophosphamide-related cardiomyopathy. When analyzing adverse events and GC-related toxicity, there was a higher event rate within the initial 3 months from diagnosis, suggesting that higher peak GC exposure may be associated with adverse events.

## Discussion

It is increasingly recognized that the use of lower dose, oral GC is feasible in the management of AAV and that further research assessing the role of IV MTP is needed. The use of IV MTP as part of remission induction therapy is widely adopted; however, its toxic effects and contribution to the cumulative burden of GC exposure may often be overlooked with a limited evidence base supporting its use.^8^ In the high-dose group over 25% of the cumulative GC dose was due to IV MTP. The dose burden of MTP has not been addressed by any of the recent steroid-sparing trials.

Our study demonstrates no significant difference in treatment outcomes, including mortality, ESKD, or relapse rates, when using a reduced dose of IV MTP and lower overall cumulative dose of GC compared with standard practice. This was observed despite the presence of more advanced renal impairment, aggressive disease on histology, and a higher RRS at presentation in the low-dose group.

While landmark trials, such as Plasma Exchange and Glucocorticoids in Severe ANCA-Associated Vasculitis,^17^ support a reduced dosing strategy of oral GC in AAV, the protocol still included high doses of IV MTP ranging between 1 and 3 g. Other trials, such as the Low-Dose Glucocorticoid Vasculitis Induction study,^18^ compared a low and high oral GC dosing regimen (0.5  versus 1 mg/kg daily) alongside rituximab, in the absence of IV MTP at induction. While this was only used in patients with nonsevere disease, this study and the Plasma Exchange and Glucocorticoids in Severe ANCA-Associated Vasculitis study demonstrated noninferiority of reduced GC treatment and potential reductions in the incidence of severe infection.^17,19^ Given there was no additional therapeutic advantage of high versus low-dose GCs with noninferiority, it suggests that the key determinant when comparing outcomes in our study was MTP dosing and overall GC burden.

Other retrospective studies have presented a feasible strategy of IV MTP avoidance alongside a reduced oral dosing regimen. McGovern *et al.*^20^ demonstrated no adverse outcomes in a cohort of 83 patients older than 65 years, all of whom were treated with low-dose oral GC, and with the exception of one, none received IV MTP. While this study lacked a control group for comparative analysis of MTP dosing as a determining variable, it demonstrated that GCs can be safely reduced with no significant effect on patient outcomes.^20^ In addition, Chanouzas *et al.*^21^ *and* McGregor *et al.*^22^ undertook studies looking at outcomes according to MTP use in the context of high-dose oral GCs. Chanouzas *et al.*^21^ did not identify any benefit of IV MTP on patient survival, renal survival, or 12-month relapse rates, but did confirm a higher rate of treatment-related adverse effects, including infection and diabetes.^21^ McGregor *et al.*^22^ identified similar findings in a subgroup analysis of 101 patients who received MTP and 46 who did not. These studies add to a growing body of evidence that supports a reduction in IV MTP and oral GC dosing.

Our study did not demonstrate a statistically significant difference in adverse events with reduced GC dosing. The reasons for this may be the small sample size limiting the statistical power and the use of retrospective data collection, which can underestimate adverse events. Despite this, the use of high-dose and cumulative GC exposure has also been explored in other conditions and is well-recognized to pose a significant risk to patients.^7,23–27^ In addition, lower dose GC therapy has other benefits, including a reduced financial burden on health care because lower quantities of treatment and reduced monitoring are needed as well as improved adherence.^28,29^

Additional studies have shown that the mortality associated with AAV is highest in the first 12 months from diagnosis.^30^ Reasons for this include the risk of infection, active vasculitis, and cardiovascular disease.^3,30,31^ Massicotte-Azarniouch *et al.*^31^ demonstrated that cardiovascular events in AAV occurred predominantly in the first 3 months from diagnosis, which is when the GC burden is highest, causing adverse effects on BP, cholesterol, and glucose tolerance.^23,24^ It is also worth noting that in addition to physical side effects, high-dose GC has been associated with significant emotional, psychological, and social effects, including high levels of depression and anxiety, as well as has been negatively affecting patients relationships, appearance, and sleep.^32^ GC reduction, therefore, should be a priority when addressing patient-reported outcomes in the management of AAV. The recent ADVOCATE trial^33^ has shown great promise with avacopan, which has had a positive effect on quality-of-life outcomes and looks to facilitate future radical steroid avoidance strategies.

We also recognize the limitations of this study, including the small sample size and use of retrospective data. The use of a historic cohort makes comparisons of immunosuppressive treatments challenging, with induction regimens using rituximab more frequently from 2009 and the potential effect of severe acute respiratory syndrome coronavirus 2 on patients from 2019 onward. Despite this, the study duration is similar to that used in other comparable studies. Induction therapy with cyclophosphamide and rituximab was slightly heterogeneous, and the cohorts were separated historically and not randomized, but other aspects of care were comparable, with the only significant difference between the groups being the GC dosing.

In conclusion, our data demonstrate that a markedly reduced dose of IV MTP with a lower overall cumulative dose of GC is safe and effective in the management of severe AAV disease, with no significant difference in primary outcomes. We suggest this be a platform for larger studies looking at reduced/no IV MTP in the management of AAV alongside significantly lower cumulative GC dosing.

## Supplementary Material

## Supplemental Material

Supplemental Table 1. Dosing for oral glucocorticoids in the high and low-dose glucocorticoid cohorts.

Supplemental Table 2. Remission induction immunosuppression, daily GC dosing, and the cumulative GC doses across the high and low-dose cohorts.
