# Supplementary material for: Low-Dose Intravenous Methylprednisolone in Remission Induction Therapy for ANCA-Associated Vasculitis
Source: Kidney360. 2023 Sep 5;4(9):e1286–92. doi: 10.34067/KID.0000000000000222 (PMC10547218; doi:10.34067/KID.0000000000000222)
Supplement: Supplementary file 1 [file kidney360-4-e1286-s001.pdf]

Supplementary Materials

Supplementary Table S1 - Dosing for oral glucocorticoids in the high and low dose glucocorticoid cohort

Week	High Dose regime	Low Dose regime
0-2	40 - 60mg OD	30 mg OD
2-4	40 - 30 mg OD	20 mg OD
4-6	25 mg OD	15 mg OD
6-8	20 mg OD	12.5 mg OD
8-10	15 mg OD	10 mg OD
10-36	10 - 5 mg OD	5 mg OD
36-40	5 mg OD	4 mg OD
40-44	5 mg OD	3 mg OD
44-48	5 mg OD	2 mg OD
48-52	5 mg OD	1 mg OD
52 +	Review	STOP

Low-dose GC (250mg IV methylprednisolone followed by a tapering course of 30mg prednisolone daily), High-dose GC (1.5g IV methylprednisolone followed by tapering course of 40-60mg prednisolone daily), OD; Once daily, mg; milligrams

Supplementary Table S2 – Remission Induction Immunosuppression, daily GC dosing and the cumulative GC doses across the high and low dose cohort

	High Dose GC (n=34)	Low dose GC (n=31)
**Remission Induction Immunosuppression**		
IV cyclophosphamide monotherapy, (n)	25	14
Rituximab monotherapy, (n)	3	8
Combination therapy, (n)	6	9
Plasma exchange, (n)	13	11
**Glucocorticoid Treatment**		
Daily GC dose at 6 months, mean ± SD (mg)	10.1 ± 5.5	6.0 ± 2.8
Daily GC dose at 12 months, mean ± SD (mg)	5.4 ± 2.5	3.9 ± 2.1
Cumulative GC dose at 3 months, mean ± SD (mg)	3687 ± 854	1644 ± 310
Cumulative GC dose at 6 months, mean ± SD (mg)	4558 ± 972	2162 ± 374
Cumulative GC dose at 12 months, mean ± SD (mg)	5604 ± 1168	2893 ± 536

GC; Glucocorticoid, IV; intravenous, mg; milligrams, *SD; standard deviation*
